# Inequities by race and ethnicity in cancer treatment receipt among people living with HIV and cancer in the U.S. (2004–2020)

**DOI:** 10.1186/s12885-025-14272-z

**Published:** 2025-05-20

**Authors:** Jessica Y. Islam, Yi Guo, Jennifer K. McGee-Avila, Kea Turner, Amir Alishahi Tabriz, Yu Chen Lin, Susan T. Vadaparampil, Anna E. Coghill, Marlene Camacho-Rivera, Gita Suneja

**Affiliations:** 1 Center for Immunization and Infection in Cancer, H. Lee Moffitt Cancer Center and Research Institute, 12902 Magnolia Drive, Tampa, FL 33612 USA; 2 Department of Cancer Epidemiology, H. Lee Moffitt Cancer Center and Research Institute, Tampa, FL USA; 3 Department of Oncologic Sciences, University of South Florida, Tampa, FL USA; 4 Department of Health Outcomes and Biomedical Informatics, College of Medicine, University of Florida, Gainesville, FL USA; 5 Infections and Immunoepidemiology Branch, Division of Cancer Epidemiology & Genetics, National Cancer Institute, Rockville, MD USA; 6 Department of Health Outcomes and Behavior, H. Lee Moffitt Cancer Center and Research Institute, Tampa, FL USA; 7 Department of Community Health Sciences, School of Public Health, SUNY Downstate Health Sciences University, New York, NY USA; 8 Department of Radiation Oncology, Huntsman Cancer Institute, University of Utah, Salt Lake City, UT USA

**Keywords:** Cancer treatment, Immunotherapy, Systemic therapy, HIV, Infectious diseases, Inequities, Quality of care

## Abstract

**Objective:**

People with HIV (PWH) are less likely to receive cancer treatment compared to those without HIV. Social factors, such as cancer treatment facility type, which may impact cancer treatment receipt among PWH have not been quantitatively explored. Our objective was to characterize racial differences in social determinants of health (SDoH) that impact cancer treatment receipt among PWH and cancer in the US.

**Methods:**

We used the National Cancer Database (2004–2020) and included adult (18–89 years) patients living with HIV identified using ICD9 and ICD10 codes. We included the 14 most common cancers that occur among PWH. Our main outcome was receipt of first-line cancer treatment, including systemic therapy, surgery, hormone therapy, and radiotherapy. Our main SDoH exposures included (1) area-level education or % of adults without a high school degree and (2) area-level income or median income quartiles within the patient’s zip code. Healthcare access measures we evaluated included insurance status, distance to care, and cancer care facility type. We used hierarchical multivariable logistic regression models to estimate adjusted logistic ratios (aOR) with 95% confidence intervals (95% CI).

**Results:**

We included 31,549 patients with HIV and cancer, of which 16% did not receive treatment. Overall, 43% of patients were aged ≥ 60 years, 38% were NH-Black, 68% were male, and 39% of patients resided in the South. 47% of patients were diagnosed with stage I/II cancer, and the most common cancers included were lung (21%), diffuse large B-cell lymphoma (12%), colorectal (9%) cancers, and prostate(9%). Compared to those in the highest quartile (Q4), PWH in the lower quartiles of educational attainment were less likely to receive cancer treatment (Q1 vs. Q4: aOR:0.74; 95% CI:0.66–0.82). Residing in the lower quartiles of household income was also inversely associated with cancer treatment receipt (Q1 vs.Q4: aOR:0.73; 95% CI:0.65–0.82). These associations were consistent among NH-White and NH-Black PWH. PWH living within 2 miles of their cancer care facility (vs. >45 miles away) and those treated at community cancer programs (vs. an academic/research program) were less likely to receive cancer treatment.

**Conclusion:**

Area-level markers of social disadvantage are associated with cancer treatment receipt among PWH, suggesting SDoH factors may impact inequities in cancer treatment by HIV status.

**Supplementary Information:**

The online version contains supplementary material available at 10.1186/s12885-025-14272-z.

## Introduction

Cancer is a leading cause of morbidity and mortality among U.S. people with HIV (PWH). Compared to those without HIV, PWH experience elevated cancer-specific mortality associated with multiple cancers, such as lung, breast, and prostate cancers [1]. Although inequities in cancer outcomes among PWH are likely multifactorial, (e.g., lower screening rates [2, 3], later-stage at diagnosis [4, 5], and differences in HIV-related tumor behavior [6]), inequities in high-quality cancer treatment are a major contributor to worse cancer outcomes among PWH [7–9].

In the U.S, several studies have demonstrated that PWH are less likely to receive cancer treatment compared to those without HIV [10]. The earliest study published in 2013 demonstrated that PWH with non-small cell lung cancer were less likely to be administered cancer treatment compared to those without HIV [11]. Data from the national HIV/AIDS Cancer Match Study demonstrated that between 2001 and 2019, PWH were almost 40% more likely to not receive cancer treatment compared to those without HIV, even after considering important factors such as stage at diagnosis, sex, age and cancer type. Further, these cancer treatment inequities in PWH experience persisted in 2014–2019 calendar years across multiple cancer sites [12].

Inequities in cancer treatment delivery are associated with multilevel factors at the individual-, healthcare systems, and societal level [13] as has been demonstrated in multiple contexts, such as by race and ethnicity in the general US population [14–17], and across multiple cancer sites [18]. In the context of living with HIV, it is important to consider racialized inequities in cancer care delivery, given the known barriers to receipt of HIV care Black and Latinx PWH experience [19–21]. To improve delivery of high-quality cancer treatment to PWH from marginalized populations, insights into potential mechanisms are needed to facilitate intervention development and ultimately eradicate inequities in cancer outcomes [22]. Our objective was to examine the role of social determinants of health (SDoH) and healthcare access measures on cancer treatment delivery among PWH by race/ethnicity.

Acknowledging race and ethnicity are social constructs [23], our objective extends beyond describing racial inequities to identify potential pathways for inequity-reduction intervention development to achieve cancer treatment equity among PWH in the US. We hypothesize that minoritized PWH will be less likely to receive cancer treatment compared to NH-White adults living with HIV, and markers of poverty or socioeconomic disadvantage (e.g., living without insurance) are likely associated with lower cancer treatment uptake. We therefore further stratified analyses by healthcare access factors including insurance status, distance to care, and cancer treatment facility type. To our knowledge, this is the first investigation of racial inequities in cancer treatment among PWH, despite the established disproportionate burden of HIV among minoritized and marginalized populations in the US [21].

## Methods

### Data source

The U.S. National Cancer Database(NCDB) is a hospital-based cancer registry of approximately 40 million records jointly sponsored by the American College of Surgeons and the American Cancer Society, which captures approximately 72% of all U.S. cancer cases from more than 1,500 facilities accredited by the American College of Surgeons’ Commission on Cancer (CoC) [24]. To maintain CoC accreditation, all facilities are mandated to report all diagnosed cancer cases to the NCDB [24]. The NCDB collects data from cancer patients engaged in first-course treatment in cancer care and surveillance including up to 1.5 million new cases of cancer each year. To ensure high-quality and accurate data, the data is standardized according to national standards and CoC-accredited sites undergo an external review of hospital charts and registry abstracts of at least 10% of records every 3 years [25].

### Study cohort

We identified people with cancer diagnosed between 2004 and 2020 with a comorbid HIV diagnosis. HIV status was determined using the ICD-9-CM diagnosis codes 04200 − 044.90, 07593, V0800 and ICD-10-CM codes B20-B22, B24, Z21. We focused on the 14 most common cancers [26] among PWH including, cancers of the head and neck (oral cavity, pharynx, and larynx), upper gastrointestinal tract (pancreas, stomach, small intestine, bile duct, gall bladder, and esophagus), colorectum, anus, lung, female breast, cervix, female genital cancers (uterus, vulva, and vagina), liver, kidney and bladder, prostate, Hodgkin lymphoma (HL); and diffuse large B-cell lymphoma (DLBCL). Cancer types were identified using the Surveillance, Epidemiology, and End Results (SEER) cancer statistics review using International Classification of Diseases for Oncology, 3rd Edition (ICD-O-3) site and histology codes [27], which are summarized in Supplementary Table 1. Cancer stage was categorized according to the American Joint Committee on Cancer staging.

### Measures

The primary outcome was receipt of first-line cancer treatment, based on data abstracted from the patients’ medical records submitted by the reporting facility. The first course of cancer treatment was ascertained for DLBCL and Hodgkin lymphoma as chemotherapy, immunotherapy, transplant (bone marrow or stem cell), radiotherapy, or a combination of all treatment types. For all other cancer types, cancer treatment was defined as surgery, radiotherapy, chemotherapy, immunotherapy, or any combination of these therapies. For prostate and breast cancers, hormone therapy was also included in the outcome definition.

We considered the only two zip-code level socioeconomic status exposure measures available in NCDB, specifically (1) the percent of adults without a high school degree (herein referred to as zip-code level educational attainment), and (2) median household income of adults aged 25 years and above (herein referred to as zip-code level income) living within a patient’s residential zip code. Both zip-code level exposures were derived using data from the American Community Survey [28] and summarized into quartiles based on the distribution of each measure among all patients in the NCDB across calendar year periods (Supplementary Table 2).

We considered three healthcare access measures available in the NCDB. First, insurance status was determined according to coding for primary payer at diagnosis and was categorized as Private, Medicaid, Medicare, Uninsured, or Other Government insurance, which could include Veteran’s Affairs insurance, Indian Health Services, etc. Second, cancer treatment facility types were defined by the CoC accreditation program and categorized as Community Cancer Program, Comprehensive Community Cancer Program, Teaching/Academic Research Program (includes NCI-designated comprehensive cancer centers), and Integrated Network Cancer Program (INCP). Third, distance between the patient’s residence (based on patient zip code or city if zip code was unavailable) and the hospital location (based on the street address for the facility) that reported the case.

To understand how area-level SDoH and healthcare access measures may differ across racialized experiences of cancer patients with HIV within healthcare systems [29], we examined differences in SDoH within various racial and ethnic groups defined as non-Hispanic (NH) White, NH-Black, Hispanic/Latinx, NH-Asian, NH-American Indian, NH-Pacific Islander, and other. Other was defined by the NCDB as mixed race or other racial categories. PWH who were NH-American Indian (*N* = 33), NH-Pacific Islander (*n* = 248), and other (*n* = 184) were excluded from the main analyses due to limited observations to conduct multi-level regression analyses yet are included in Table 1 for descriptive purposes.

**Table 1:** Sociodemographic and cancer clinical characteristics of persons living with HIV and cancer in the National Cancer database by race and ethnicity (*n* = 31,549; 2004–2020)

					Racial and Ethnic Groups											
	Total		Received Curative Cancer Treatment		NH-Asian (*n* = 246)		NH-American Indian (*n* = 81)		NH-Black (*n* = 11834)		Hispanic/Latinx (*n* = 3038)		NH-Pacific Islander (*n* = 32)		NH-White (*n* = 15920)	
	No.	Col %	No.	Row %	No.	Col %	No.	Col %	No.	Col %	No.	Col %	No.	Col %	No.	Col %
Race and ethnicity																
NH-Asian	246	0.8	208	84.6												
NH-American Indian	81	0.3	73	90.1												
NH-Black	11,834	37.5	9708	82.0												
Hispanic/Latinx	3038	9.6	2535	83.4												
NH-Pacific Islander	32	0.1	29	90.6												
NH-White	15,920	50.5	13,480	84.7												
Other	183	0.6	152	83.1												
Missing	215	0.7	168													
Age Groups (Years)																
<40	2349	7.4	2084	88.7	31	12.6	5	6.2	1250	10.6	383	12.6	2	6.3	641	4.0
40–49	5831	18.5	5030	86.3	51	20.7	19	23.5	2570	21.7	775	25.5	10	31.3	2323	14.6
50–59	9746	30.9	8199	84.1	67	27.2	29	35.8	4250	35.9	997	32.8	13	40.6	4284	26.9
60+	13,623	43.2	11,040	81.1	97	39.4	28	34.6	3764	31.8	883	29.1	7	21.9	8672	54.5
Sex																
Male	21,579	68.4	17,894	83.0	168	68.3	56	69.1	7566	63.9	2251	74.1	24	75.0	11,229	70.5
Female	9970	31.6	8459	84.9	78	31.7	25	30.9	4268	36.1	787	25.9	8	25.0	4691	29.5
Patient’s Census Region																
Northeast	7941	25.2	6535	82.3	66	26.8	8	9.9	2782	23.5	1127	37.1	2	6.3	3750	23.6
South	12,402	39.3	10,224	82.5	46	18.7	25	30.9	5926	50.1	914	30.1	4	12.5	5423	34.1
Midwest	5047	16.0	4267	84.5	15	6.1	12	14.8	1380	11.7	164	5.4	0	0.0	3421	21.5
West	3810	12.1	3243	85.1	88	35.8	31	38.3	496	4.2	450	14.8	24	75.0	2685	16.9
Missing†	2349	7.4	2084		31	12.6	5	6.2	1250	10.6	383	12.6	2	6.3	641	4.0
Area of Residence																
Urban	30,498	96.7	25,442	83.4	236	95.9	71	87.7	11,577	97.8	2995	98.6	31	96.9	15,205	95.5
Rural	263	0.8	222	84.4	0	0.0	5	6.2	60	0.5	5	0.2	0	0.0	191	1.2
Missing	788	2.5	689		10	4.1	5	6.2	197	1.7	38	1.3	1	3.1	524	3.3
Distance from patient to provider																
<2 miles	4504	14.3	3579	79.5	32	13.0	11	13.6	1923	16.2	526	17.3	5	15.6	1935	12.2
2–9 miles	13,875	44.0	11,480	82.8	133	54.1	26	32.1	5816	49.1	1394	45.9	13	40.6	6295	39.5
10–45 miles	7973	25.3	6826	85.6	52	21.1	19	23.5	2332	19.7	715	23.5	12	37.5	4776	30.0
>45 miles	2468	7.8	2090	84.7	9	3.7	18	22.2	588	5.0	130	4.3	1	3.1	1688	10.6
Missing	2729	8.7	2378		20	8.1	7	8.6	1175	9.9	273	9.0	1	3.1	1226	7.7
Percentage of adults residing without a high school degree in patient’s zip code (quartiles)‡																
Q1 (Lowest Educational Attainment)	8969	28.4	7254	80.9	62	25.2	16	19.8	4654	39.3	1544	50.8	8	25.0	2552	16.0
Q2	8009	25.4	6669	83.3	60	24.4	27	33.3	3442	29.1	594	19.6	7	21.9	3773	23.7
Q3	6183	19.6	5237	84.7	44	17.9	15	18.5	1668	14.1	371	12.2	7	21.9	4018	25.2
Q4 (Highest Educational Attainment)	5339	16.9	4555	85.3	60	24.4	15	18.5	804	6.8	238	7.8	9	28.1	4143	26.0
Missing	3049	9.7	2638		20	8.1	8	9.9	1266	10.7	291	9.6	1	3.1	1434	9.0
Median household income based on patient’s zip code‡																
Q1 (Lowest Median Income)	8341	26.4	6728	80.7	26	10.6	21	25.9	4968	42.0	996	32.8	3	9.4	2217	13.9
Q2	6129	19.4	5089	83.0	33	13.4	17	21.0	2222	18.8	625	20.6	4	12.5	3168	19.9
Q3	6374	20.2	5364	84.2	64	26.0	16	19.8	1849	15.6	570	18.8	10	31.3	3786	23.8
Q4 (Highest Median Income)	7643	24.2	6526	85.4	102	41.5	19	23.5	1521	12.9	555	18.3	14	43.8	5312	33.4
Missing	3062	9.7	2646		21	8.5	8	9.9	1274	10.8	292	9.6	1	3.1	1437	9.0
Insurance Type or Primary Payor																
Uninsured	1720	5.5	1340	77.9	20	8.1	4	4.9	829	7.0	311	10.2	0	0.0	531	3.3
Privately Insured	8956	28.4	7949	88.8	83	33.7	12	14.8	3041	25.7	770	25.3	13	40.6	4917	30.9
Medicaid	6633	21.0	5391	81.3	54	22.0	20	24.7	3702	31.3	994	32.7	8	25.0	1741	10.9
Medicare	13,571	43.0	11,154	82.2	85	34.6	38	46.9	3970	33.5	902	29.7	11	34.4	8434	53.0
Other Government	305	1.0	252	82.6	0	0.0	7	8.6	128	1.1	19	0.6	0	0.0	148	0.9
Missing	364	1.2	267		4	1.6	0	0.0	164	1.4	42	1.4	0	0.0	149	0.9
Cancer Care Facility Type																
Community Cancer Program	1379	4.4	1034	75.0	6	2.4	3	3.7	297	2.5	72	2.4	2	6.3	989	6.2
Comprehensive Community Cancer Program	8638	27.4	6996	81.0	53	21.5	24	29.6	2108	17.8	546	18.0	10	31.3	5854	36.8
Academic/Research Program	13,992	44.4	11,881	84.9	128	52.0	44	54.3	6235	52.7	1571	51.7	18	56.3	5775	36.3
Integrated Network Cancer Program	5191	16.5	4358	84.0	28	11.4	5	6.2	1944	16.4	466	15.3	0	0.0	2661	16.7
Missing†	2349	7.4	2084		31	12.6	5	6.2	1250	10.6	383	12.6	2	6.3	641	4.0
Year of Cancer Diagnosis																
2004–2009	12,708	40.3	10,445	82.2	87	35.4	25	30.9	3367	28.5	902	29.7	9	28.1	8171	51.3
2010–2015	7322	23.2	6038	82.5	63	25.6	22	27.2	3096	26.2	831	27.4	6	18.8	3214	20.2
2016–2020	11,519	36.5	9870	85.7	96	39.0	34	42.0	5371	45.4	1305	43.0	17	53.1	4535	28.5
Cancer Type																
Lung	6460	20.5	4548	70.4	35	14.2	13	16.0	2342	19.8	345	11.4	5	15.6	3651	22.9
DLBCL	3675	11.6	3196	87.0	45	18.3	13	16.0	1443	12.2	606	19.9	8	25.0	1497	9.4
Colorectal	2967	9.4	2762	93.1	24	9.8	8	9.9	786	6.6	217	7.1	3	9.4	1890	11.9
Prostate	2942	9.3	2479	84.3	20	8.1	5	6.2	1283	10.8	211	6.9	2	6.3	1387	8.7
Anus	2672	8.5	2554	95.6	10	4.1	13	16.0	967	8.2	260	8.6	3	9.4	1391	8.7
Head & Neck	2213	7.0	2045	92.4	14	5.7	5	6.2	739	6.2	221	7.3	2	6.3	1210	7.6
Upper GI	2222	7.0	1526	68.7	17	6.9	5	6.2	801	6.8	212	7	1	3.1	1156	7.3
Breast	1982	6.3	1902	96.0	22	8.9	2	2.5	878	7.4	142	4.7	0	0.0	917	5.8
Kidney and bladder	1885	6.0	1675	88.9	12	4.9	5	6.2	552	4.7	144	4.7	1	3.1	1151	7.2
HL	1442	4.6	1245	86.3	11	4.5	2	2.5	665	5.6	282	9.3	0	0.0	455	2.9
Liver	1462	4.6	931	63.7	20	8.1	5	6.2	613	5.2	252	8.3	5	15.6	543	3.4
Female genital cancers	1109	3.5	1029	92.8	9	3.7	2	2.5	420	3.5	82	2.7	2	6.3	582	3.7
Cervix	518	1.6	461	89.0	7	2.8	3	3.7	345	2.9	64	2.1	0	0.0	90	0.6
Stage at Diagnosis																
Stage 1	7914	25.1	6961	88.0	69	28.0	28	34.6	2719	23.0	761	25	7	21.9	4242	26.6
Stage 2	6886	21.8	6248	90.7	54	22.0	17	21.0	2436	20.6	611	20.1	6	18.8	3686	23.2
Stage 3	6629	21.0	5723	86.3	46	18.7	20	24.7	2570	21.7	601	19.8	6	18.8	3307	20.8
Stage 4	10,120	32.1	7421	73.3	77	31.3	16	19.8	4109	34.7	1065	35.1	13	40.6	4685	29.4
Cancer Treatment Type*																
Surgery	13,693	43.4			98	39.8	34	42.0	4458	37.7	1130	37.2	14	43.8	7807	49.0
Radiation	9949	31.5			79	32.1	45	55.6	3990	33.7	867	28.5	11	34.4	4845	30.4
Chemotherapy	13,977	44.3			125	50.8	37	45.7	5468	46.2	1595	52.5	18	56.3	6568	41.3
Hormone	3830	12.1			33	13.4	10	12.3	1616	13.7	451	14.8	8	25.0	1672	10.5
Immunotherapy	1885	6.0			23	9.3	6	7.4	783	6.6	240	7.9	5	15.6	796	5.0
Transplant	32	0.1			0	0.0	1	1.2	10	0.1	2	0.1	0	0.0	17	0.1

*Treatment status is missing for 7 people

† Missing for those aged 40 years and below

### Statistical analysis

We descriptively summarized patient sociodemographic and clinical cancer characteristics as percentages overall and among those who received first-line cancer treatment using chi-square tests. Using multi-level logistic regression, we calculated adjusted odds ratios (aOR) with 95% confidence intervals to assess the association of area-level education and income with receipt of cancer treatment overall and by race/ethnicity. Next, we evaluated the associations of each healthcare access measure with cancer treatment receipt overall and by race/ethnicity among PWH. For each model, we calculated cluster-robust standard errors to account for non-independence within clusters at the facility level (i.e., to adjust for correlated patient characteristics within hospitals). All models were adjusted for age, sex, stage at cancer diagnosis, year of cancer diagnosis, and cancer type. Due to the exploratory nature of this analysis, we did not include an adjustment for multiple comparisons [30]. All analyses were performed with Stata statistical software, version 15.0 (StataCorp).

## Results

### Sociodemographic characteristics

Overall, we included 31,549 PWH with a cancer that was diagnosed between 2004 and 2020. The study population included 68% men, 47% NH-Black or Hispanic/Latinx, 39% resided in the Southern census region, and 97% resided in urban areas of the US (Table 1). The median age of PWH in this study was 57 years (interquartile range or IQR: 49–67 years). Approximately 14% resided less than 2 miles away from their oncologist; the median distance between a patient and their provider was 6.5 miles (IQR: 2.9–15.1). Overall, 21% were insured through Medicaid, 43% were on Medicare, 28% were privately insured and about 6% were uninsured. 44% were treated at an academic/research program. The most common cancers included lung (21%), DLBCL (12%), colorectal (9%) and prostate (9.2%) cancers.

### Treatment use patterns, Racial/Ethnic inequities and Area-level social determinants of health

Overall, 83.6% of PWH received first-line cancer treatment. Among patients who received treatment, 43% received surgery, 44% received chemotherapy, 12% received hormone therapy, and 31% received radiation (Table 1). After adjustment for age, sex, year of cancer diagnosis, cancer stage at diagnosis, and cancer type, we found that NH-Black PWH (aOR: 0.78; 95% CI: 0.71–0.84) and Hispanic/Latinx PWH (aOR: 0.82; 95% CI: 0.72–0.95) were significantly less likely compared to NH-White PWH to receive cancer treatment. No significant differences were observed among NH-AI (aOR: 1.35; 95% CI: 0.61–3.01), NH-Pacific Islander (aOR: 1.91; 95% CI: 0.61–1.37), or NH-Asian (aOR: 0.91; 95% CI: 0.61–1.37) PWH. In Supplementary Fig. 1, we summarized racial and ethnic differences in cancer treatment receipt among PWH by cancer site. Overall, when compared to NH-White PWH, we observed the most significant racial and ethnic differences among Black PWH with head and neck cancers, colorectal cancer, kidney and bladder cancers, and DLBCL.

**Fig. 1:**
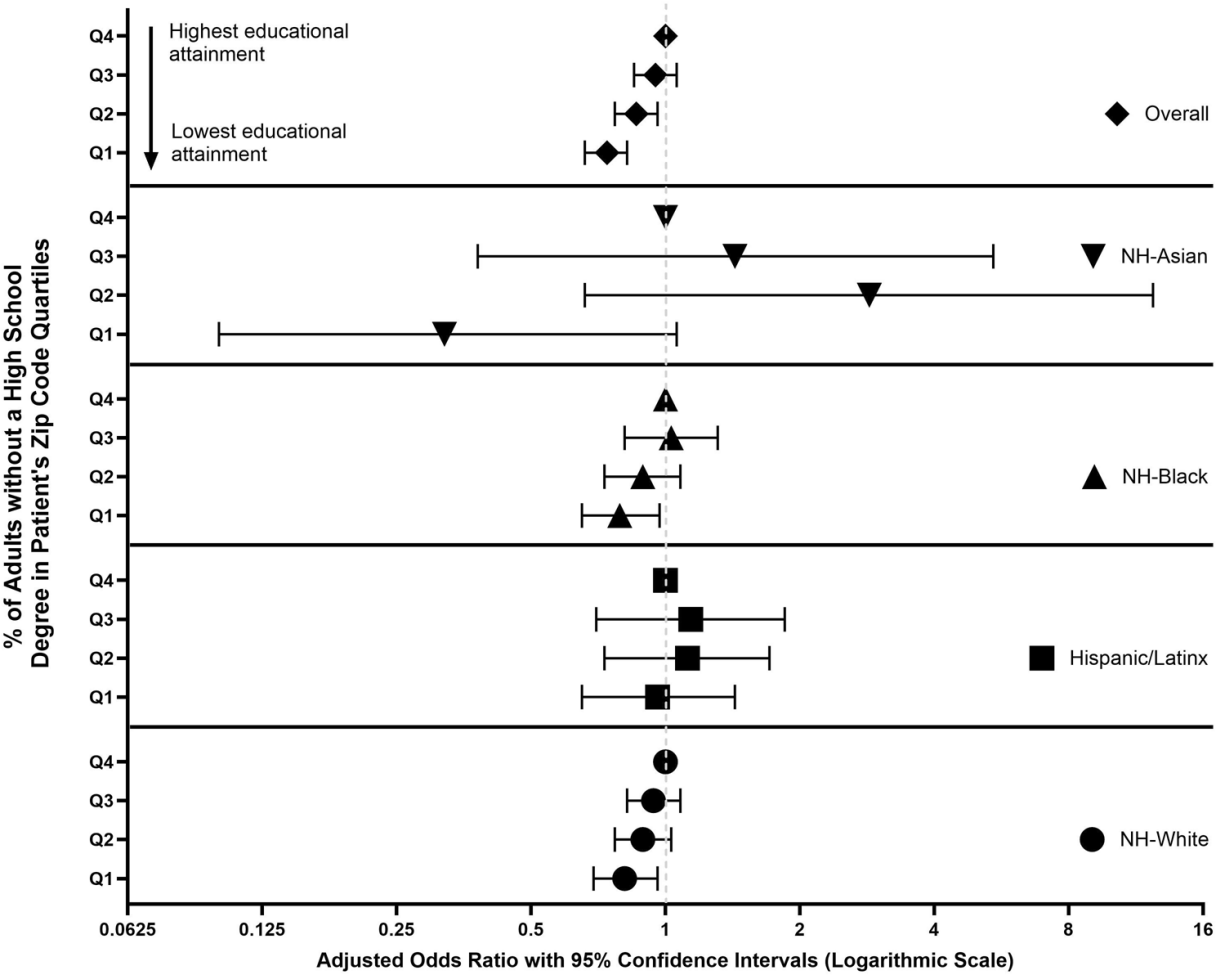
Associations of zip code level quartiles of % of adults without a high school degree residing in a patient’s zip code (i.e., educational attainment) with cancer treatment receipt among people with HIV and cancer overall and by race/ethnicity, National Cancer Database (2004–2020)

**Fig. 2:**
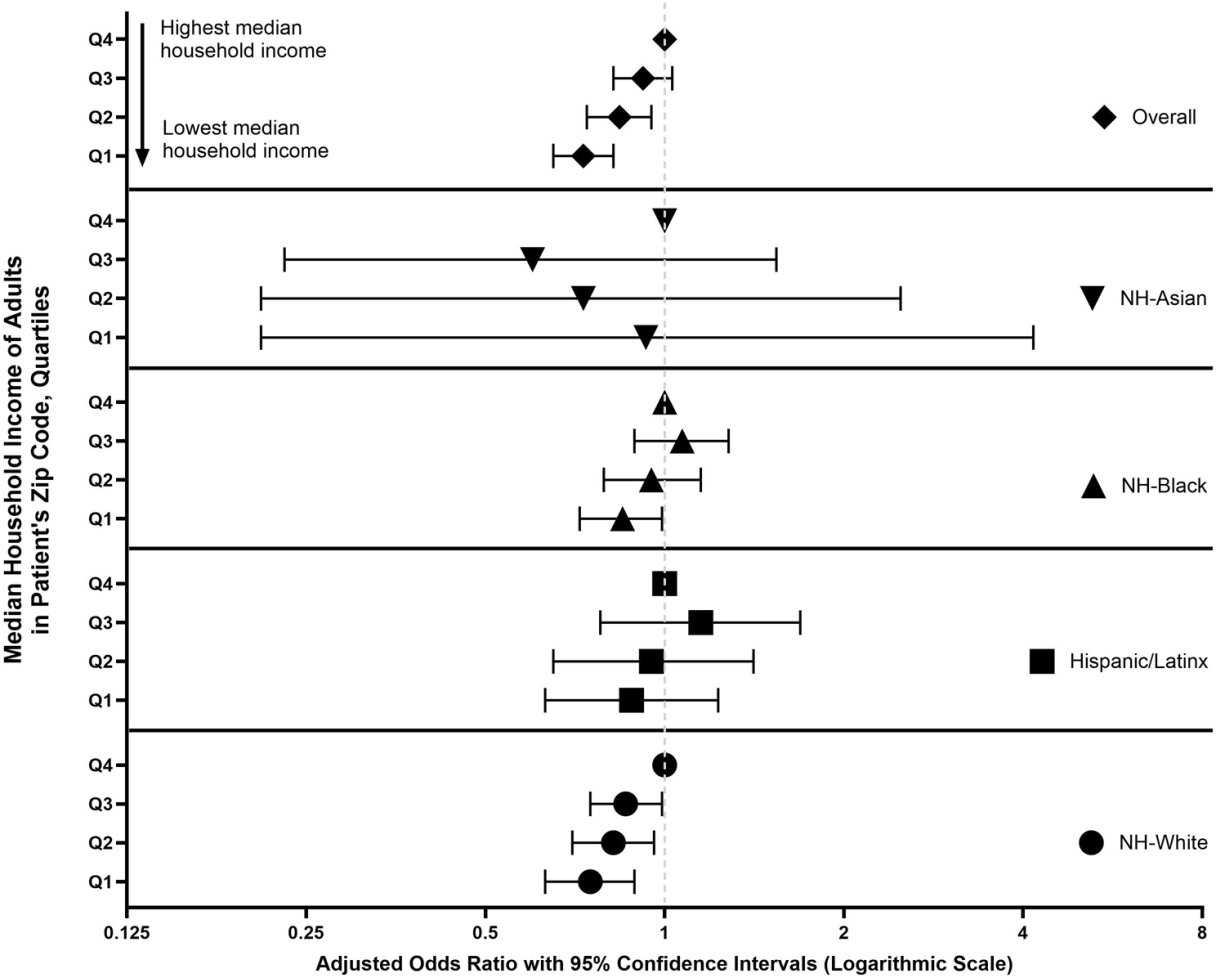
Associations of zip code level quartiles of median household income of adults residing in a patient’s zip code (i.e., educational attainment) with cancer treatment receipt among people with HIV and cancer overall and by race/ethnicity, National Cancer Database (2004–2020)

Figure 1 summarizes associations between cancer treatment receipt with zip-code level educational attainment by race and ethnicity. When we look at the estimates overall, we observe that compared to those in the highest quartile (Q4), PWH in the lower quartiles were less likely to receive cancer treatment (e.g., Q1 vs. Q4 aOR:0.74; 95% CI: 0.66–0.82). When we stratified by race/ethnicity, we did not observe associations of area-level SDoH with cancer treatment receipt among NH-Asian or Hispanic/Latinx PWH. Among NH-White (Q1 vs. Q4: aOR: 0.81; 95% CI: 0.69–0.96) and NH-Black (Q1 vs. Q4; 95% CI: 0.79; 95% CI: 0.65–0.97) PWH, residing in the lower quartiles of zip code level educational attainment was negatively associated with cancer treatment receipt (see Fig. 2).

We observed similar trends when we evaluated zip code level income. Overall, residing in the zip codes characterized by lower quartiles of household income was inversely associated with cancer treatment receipt compared to residing in a higher-income zip code (Q1 vs. Q4: aOR: 0.73; 95% CI: 0.65–0.82). Among NH-Black PWH, those residing in the lowest income quartiles had 15% lower odds of receiving cancer treatment (Q1 vs. Q4: aOR: 0.85; 95% CI: 0.72–0.99). Among NH-White PWH, residing in a lower income zip code was also inversely associated with cancer treatment receipt (Q1 vs. Q4: aOR: 0.75; 95% CI: 0.63–0.89; Q2 vs. Q4: aOR: 0.82; 95% CI: 0.70–0.96). No associations between zip code level income and cancer treatment receipt were observed among Hispanic/Latinx or NH-Asian PWH.

### Healthcare access measures by race and ethnicity

Overall, compared to PWH with Medicare insurance, PWH with private insurance were more likely to receive cancer treatment (aOR: 1.44; 95% CI: 1.32–1.57); in contrast, PWH with Medicaid (aOR: 0.84; 95% CI: 0.76–0.91) and without insurance (aOR: 0.63; 95% CI: 0.54–0.73) were less likely to receive treatment compared to Medicare recipients. These patterns were consistent among NH-Black and NH-White PWH (Table 2). Compared to PWH who received care at an academic/research program, PWH receiving care at a community cancer program (aOR: 0.56; 95% CI: 0.47–0.66) and a comprehensive community cancer program (aOR:0.77; 95% CI:0.67–0.89) were less likely to receive cancer treatment (Table 3). We observed consistent results among NH-White, NH-Black, and Hispanic/Latinx PWH. In addition, Hispanic/Latinx PWH treated at an INCP were also less likely to receive cancer treatment (aOR: 0.73; 95% CI:0.55–0.97). No associations were observed among NH-Asian PWH.

**Table 2:** Associations of insurance status with cancer treatment receipt among people with HIV and cancer overall and by race/ethnicity, National Cancer database (2004–2020)

	Overall		NH-Asian		NH-Black		Hispanic/Latinx		NH-White		
	aOR	95% CI	aOR	95% CI	aOR	95% CI	aOR	95% CI	aOR	95% CI	
Privately Insured	**1.44**	**1.32–1.57**	3.38	0.86–13.23	**1.26**	**1.09–1.46**	1.20	0.91–1.59	**1.56**	**1.42–1.79**	
Medicaid	0.84	0.76–0.91	0.77	0.20–2.97	0.88	0.78–1.01	0.90	0.68–1.18	**0.81**	**0.69–0.95**	
Medicare (Ref)	1.00		1.00		1.00		1.00		1.00		
Uninsured	0.63	0.54–0.73	0.43	0.11–1.71	**0.72**	**0.58–0.89**	0.78	0.55–1.10	**0.53**	**0.41–0.67**	
Other Government	0.98	0.72–1.33	-		1.04	0.64–1.69	1.56	0.40–1.36	0.83	0.51–1.34	

Models adjusted for sex, age group, stage at cancer diagnosis, year of cancer diagnosis, and cancer type

**Table 3:** Associations of cancer care facility type with cancer treatment receipt among people with HIV and cancer overall and by race/ethnicity, National Cancer Database (2004–2020)

	Overall		NH-Asian		NH-Black		Hispanic/Latinx		NH-White	
	aOR	95% CI	aOR	95% CI	aOR	95% CI	aOR	95% CI	aOR	95% CI
Academic/Research Program (Ref)	1.00		1.00		1.00		1.00		1.00	
Community Cancer Program	**0.56**	**0.47–0.66**	**-**		**0.57**	**0.42–0.78**	**0.47**	**0.25–0.89**	**0.47**	**0.38–0.60**
Comprehensive Community Cancer Program	**0.77**	**0.67–0.89**	0.45	0.15–1.38	0.86	0.72–1.01	**0.58**	**0.41–0.82**	**0.68**	**0.57–0.80**
Integrated Network Cancer Program	0.95	0.79–1.13	0.52	0.12–2.17	1.02	0.78–1.33	**0.73**	**0.55–0.97**	0.92	0.76–1.11

Models adjusted for sex, age group, stage at cancer diagnosis, year of cancer diagnosis, and cancer type

Note: The CoC categorizes cancer care facilities based on number of cancer cases newly diagnosed each year, teaching facilities, and structure of governance of the facility. Community and comprehensive community cancer programs differ by patient volume, with comprehensive community programs identifying more than 500 cancer cases each year. INCPs are cancer care facilities that are owned by a larger organization with integrated and comprehensive cancer care services. Academic/research programs offer postgraduate medical education with more than 500 cases of cancer each year

Overall, PWH who lived less than 2 miles (aOR:0.74; 95% CI:0.61–0.89) were less likely to receive cancer treatment compared to those who lived over 45 miles away (Table 4). When stratified by race/ethnicity, NH-White PWH living less than 2 miles from their cancer care provider had 31% lower odds of receiving cancer treatment compared to those living over 45 miles away (aOR:0.69; 95% CI:0.54–0.87).

**Table 4:** Associations of distance to care with cancer treatment receipt among people with HIV and cancer overall and by race/ethnicity, National Cancer Database (2004–2020)

	Overall		NH-Asian		NH-Black		Hispanic/Latinx		NH-White	
	aOR	95% CI	aOR	95% CI	aOR	95% CI	aOR	95% CI	aOR	95% CI
< 2 Miles	**0.74**	**0.61–0.89**	-		0.87	0.64–1.20	0.87	0.48–1.57	**0.69**	**0.54–0.87**
2–9 Miles	0.87	0.73–1.03	0.76	0.11–5.24	0.96	0.71–1.29	0.86	0.49–1.50	0.91	0.75–1.10
10–45 Miles	1.06	0.90–1.25	-		1.08	0.79–1.49	1.21	0.69–2.12	1.08	0.89–1.29
> 45 miles (Ref)	1.00		1.00		1.00		1.00		1.00	

Models adjusted for sex, age group, stage at cancer diagnosis, year of cancer diagnosis, and cancer type

Note: Residential latitude and longitude are based on the patient’s zip code centroid or on the city if the zip code was not available. Hospital locations are based on the street address for the facility. The great circle distance is calculated between those two points. In some instances, the residential city is outside of the United States, so the upper bound of distance may be quite large (highest = 3723 miles). A distance of 0 can result when the patient lives in the same zip code where the facility is located (*n* = 2).The Haversine (halfversedsine) formula is used to calculate the distance between the two locations

## Discussion

Racial inequities and differences across healthcare access, as measured via insurance status and type of cancer care facility, are highly salient in the context of living with both HIV and cancer. We demonstrate that NH-Black and Hispanic/Latinx PWH are less likely to receive cancer treatment compared to NH-White PWH. We found that living in an area with lower socioeconomic status was negatively associated with receiving cancer treatment, particularly among NH-White and NH-Black PWH. Further, our results suggest that PWH without Medicaid coverage, no insurance or those receiving care at community cancer clinics may experience additional barriers to cancer treatment. The role of SDoH and race/ethnicity in cancer care quality has been demonstrated across multiple cancer sites and contexts [13, 31]; however, this is the first investigation into the role of these factors in relation to cancer treatment receipt among PWH. Given that PWH are a growing [22], understudied [32] cancer patient population in the US with long-standing inequitable access to cancer care, translating our findings to actionable opportunities for targeted intervention development will be key to improving cancer care quality of PWH from minoritized communities.

In the present analysis, we operationalize self-reported race and ethnicity as a social construct within the context of healthcare delivery to explore differences in receipt of cancer treatment among PWH., On a population level, racial and ethnic identities can approximate access to resource distribution as a fundamental cause of poor access to care. Understanding the role of race and ethnicity in cancer care quality among PWH is an unexplored opportunity to develop inequity-reduction interventions targeting racial disparities in cancer treatment uptake among US PWH. Existing inequity-reduction interventions targeting racial disparities in other cancers are heterogenous in nature, but many rely largely on patient navigation programs led by staff nurses or social workers, frequently in tandem with coupled interventions such as enhanced follow-up measures [33–36]. For example, the Accountability for Cancer Care through Undoing Racism and Equity or ACCURE intervention to address racial disparities in treatment completion among breast and lung cancer patients included multiple strategies, specifically a real time registry derived from electronic health records of participants to signal missed appointments or unmet care milestones, health equity training for oncologists, a navigator, and clinical feedback [36]. The ACCURE intervention increased guideline-concordant care among Black patients and eliminated treatment inequities by race among their patient population. Simulation models have also demonstrated that increases in chemotherapy receipt for breast cancer treatment among minoritized patients can lead to decreases in the cancer mortality gap among Black and White cancer patients [37].

Interventions to address cancer care quality among PWH cannot be developed based on insights from the general population due to limited generalizability based on significantly different sociodemographic characteristics (e.g., race and ethnicity, age, sex distribution) of people with HIV in the US. Importantly, in the US, PWH experienced elevated marginalization with the burden of HIV largely occurring among Black or African American and Hispanic/Latinx people, and sexual and gender minorities, who are also more likely to experience food insecurity, residential instability, and limited access to healthcare or live without insurance compared to those without HIV [38–40]. To optimize care within this understudied population, targeted intervention packages should accommodate the important underlying sociodemographic and unique HIV-related clinical and social factors, such as existing HIV-related stigma and perceptions of the healthcare system that stem from clinical encounters during HIV care [41–43].

Prior research has demonstrated that PWH are less likely to receive cancer treatment compared to people without HIV across all cancer sites evaluated (except anal cancer), even after adjustment for insurance status and medical comorbidities [8]. Most recently, using updated HACM data through 2019 including 12 US states, we demonstrated that inequities in cancer treatment delivery to PWH persist in recent (2014–2019) calendar years, particularly in certain cancer sites such as lung and cervical cancers [12]. This most recent analysis was also able to investigate factors that may be associated with cancer treatment use among PWH and found that NH-Black adults, people who inject drugs, and PWH over the age of 65 years were less likely to receive cancer treatment. Beyond quantitative epidemiological studies, one qualitative study has been conducted of 27 PWH where barriers to cancer treatment were discussed. The financial burden associated with cancer treatment was an important barrier, with participants expressing concerns regarding their inability to work due to the physical and mental health symptoms associated with treatment [44]. Further accessibility issues such as transportation difficulties (long driving distance), parking availability, and long wait times were further barriers to care cited.

Health related social needs, which stem from adverse SDoH such as contextual poverty, are an important but less explored issue when investigating cancer care inequities among PWH. While our present analysis addresses an important gap in existing knowledge regarding patterns of use of cancer treatment among PWH, with a specific focus on healthcare access and contextual poverty, future work will need to be conducted to move beyond variables that are available in the NCDB data resource. For example, our analysis suggests that PWH living within 2 miles of their cancer care provider are less likely to receive cancer treatment compared to those living over 45 miles away, particularly NH-White PWH. This finding is similar to prior studies that have shown that rural cancer patients are more likely to receive cancer care and be adherent to their recommended cancer treatment regimen due to reliable access to transportation and personal vehicles [45]. This phenomenon has been coined as the urban-rural paradox. PWH who live within close distance to their cancer care providers likely reside in urban areas, where patients rely on public transport. Patient-reported measures regarding mode of transportation, costs associated with transportation, and details regarding travel patterns for in-patient care would be important for future work. Additionally, focusing on inter-categorical assessments of PWH will be valuable to assess context specific barriers for targeted interventions to improve quality of cancer care to people living with HIV with multiple identities.

PWH are recommended to receive similar cancer treatment paradigms as those without HIV, particularly those with well controlled HIV [46]. Given the NCDB is a clinical oncology database, detailed HIV related factors are not routinely collected. As such, we are unable to consider factors such as adherence to HIV treatment, CD4 count, or date of HIV diagnosis. In addition, we are unable to consider important patient level factors, such as patient decisions to receive treatment, or assess the role of quality of patient-provider communications in the cancer treatment decision process. Future work focusing on gaining further insights into the role quality of cancer treatment from the patient experience may play on cancer treatment decision making will be critical to develop interventions to address cancer treatment inequities.

In conclusion, our findings demonstrate that about one in five PWH do not receive first-line cancer treatment. We show that living in lower socioeconomic areas is a barrier to receiving equitable cancer treatment; however, this should be further explored by incorporating further area-level measures of different SDoH domains at the county or census-tract level, such as social vulnerability or the area deprivation index. Importantly, we were able to examine healthcare access factors, including cancer care facility type. Outside of the academic hospital setting, resources to provide high quality care to PWH may be limited in community settings due to multiple factors, such as lack of familiarity with guidelines regarding cancer treatment appropriate for PWH, concerns regarding patient frailty, and the potential for drug interactions between systemic therapies and HIV medications. In addition, community cancer clinics may refer patients to larger academic hospital systems due to the availability of infectious disease physicians and more specialized cancer treatments and teams. Efforts to reduce barriers to care should be made for equitable opportunity to receive cancer treatment regardless of HIV status at all cancer treatment facility types.

## Electronic supplementary material

Below is the link to the electronic supplementary material.

Supplementary Material 1

## Data Availability

The data that support the findings of this study are available on request from the corresponding author. The data are not publicly available due to privacy or ethical restrictions.
