# Supplementary material for: Inequities by race and ethnicity in cancer treatment receipt among people living with HIV and cancer in the U.S. (2004–2020)
Source: BMC Cancer. 2025 May 20;25:897. doi: 10.1186/s12885-025-14272-z (PMC12090492; doi:10.1186/s12885-025-14272-z)

| **Supplementary Table 1. International Classification of Diseases for Oncology, 3rd Edition topography and morphology codes for** | | |
| --- | --- | --- |
| **primary cancers identified among patients in the National Cancer Database, 2004 – 2020** | | |
| Cancer site | ICD-O-3 topography code | ICD-O-3 morphology codes |
| Anus, Anal Canal & Anorectum | C210 – C218 | All^a^ |
| Breast | C500 – C509 | All^a^ |
| Colon | C180 – C189, C199 | All^a^ |
| DLBCL | Any | 9680 |
| Esophagus | C150 – C159 | All^a^ |
| Gall Bladder | C239 | All^a^ |
| Hodgkin Lymphoma | Any | 9650–9667 |
| Kidney & Renal Pelvis | C649, C659 | All^a^ |
| Larynx | C320 | All^a^ |
| Lip | C00-C009 | All^a^ |
| Liver & Intrahepatic Bile Duct | C220, C21 | All^a^ |
| Lung & Bronchus | C340 – C349 | All^a^ |
| Ovary | C569 | All^a^ |
| Pancreas | C250 – C259 | All^a^ |
| Pharynx | C10, C13, C140 | All^a^ |
| Prostate | C619 | All^a^ |
| Rectum | C209 | All^a^ |
| Small Intestine | C170 – C179 | All^a^ |
| Salivary gland | C079-C089 | All^a^ |
| Stomach | C160 – C169 | All^a^ |
| Testis | C620, C621, C629 | All^a^ |
| Thyroid | C739 | All^a^ |
| Tongue | C019, C020 – C029 | All^a^ |
| Urinary Bladder | C670 – C689 | All^a^ |
| Uterine Corpus | C540 – C559 | All^a^ |
| Uterine Cervix | C530 – C531, C538 – C539 | All^a^ |
| Vagina & Other Genital, Female | C529, C570 – C579 | All^a^ |
| Vulva | C510 – C512, C518 – C519 | All^a^ |
| ^a^Cancers with the histology codes 9050 – 9055, 9140, and 9590 – 9989 were excluded | | |

| **Supplementary Table 2. Definitions of our main area-level SDoH quartiles (Q1-Q4) by patient's cancer diagnosis year** | | | | | |
| --- | --- | --- | --- | --- | --- |
| **Patient's Cancer Diagnosis Years** | **US Census Data** | **Percent without High School Degree Quartiles** | | **Median Household Income Quartiles** | |
| 2004-2007 | 2000 US Census Data | Q1 | 29.0% + | Q1 | < $30,000 |
|  |  | Q2 | 20.0% - 28.9% | Q2 | $30,000 - $34,999 |
|  |  | Q3 | 14.0%-19.9% | Q3 | $35,000 - $45,999 |
|  |  | Q4 | < 14.0% | Q4 | $46,000 + |
|  |  |  |  |  |  |
| 2008-2012 | 2012 American Community Survey Data (Spanning 2008-2012) | Q1 | 21.0% + | Q1 | < $38,000 |
|  |  | Q2 | 13.0% - 20.9% | Q2 | $38,000 - $47,999 |
|  |  | Q3 | 7.0%-12.9% | Q3 | $48,000 - $62,999 |
|  |  | Q4 | < 7.0% | Q4 | $63,000 + |
|  |  |  |  |  |  |
| 2013-2016 | 2016 American Community Survey Data (Spanning 2012-2016) | Q1 | 17.6% + | Q1 | < $40,227 |
|  |  | Q2 | 10.9% - 17.5% | Q2 | $40,227 - $50,353 |
|  |  | Q3 | 6.3% - 10.8% | Q3 | $50,354 - $63,332 |
|  |  | Q4 | < 6.3% | Q4 | $63,333 + |
|  |  |  |  |  |  |
| 2017-2020 | 2020 American Community Survey Data (Spanning 2016-2020) | Q1 | 15.3% + | Q1 | < $46,277 |
|  |  | Q2 | 9.1% - 15.2% | Q2 | $46,277 - $57,856 |
|  |  | Q3 | 5.0% - 9.0% | Q3 | $57,857 - $74,062 |
|  |  | Q4 | < 5.0% | Q4 | $74,063 + |

| **Supplementary Table 3: Characteristics of persons living with HIV and cancer in the National Cancer Database by area-level measures**  **(2004-2020)** | | | | | | | | |
| --- | --- | --- | --- | --- | --- | --- | --- | --- |
|  | **Percentage of adults residing without a high school degree in patient's zip code**  **(quartiles; n = 28500)** | | | | **Median household income based on patient's zip code**  **(quartiles; n = 28487)** | | | |
|  | **Q1 (%) (n=8969)** | **Q2 (%) (n=8009)** | **Q3 (%) (n=6183)** | **Q4 (%) (n=5339)** | **Q1 (%) (n=8341)** | **Q2 (%) (n=6129)** | **Q3 (%) (n=6374)** | **Q4 (%) (n=7643)** |
|  |  |  |  |  |  |  |  |  |
| Age Groups (Years) |  |  |  |  |  |  |  |  |
| <40 | 8.9 | 7.8 | 6.6 | 5.4 | 9.1 | 7.7 | 7.2 | 5.7 |
| 40-49 | 21 | 19.2 | 17.2 | 14.4 | 21.3 | 19.1 | 17.9 | 15.2 |
| 50-59 | 34 | 32.3 | 28.5 | 24.2 | 33.9 | 31.6 | 29.5 | 26.7 |
| 60+ | 36.1 | 40.6 | 47.7 | 56.1 | 35.7 | 41.6 | 45.5 | 52.4 |
| Sex |  |  |  |  |  |  |  |  |
| Male | 67.4 | 67.6 | 69.1 | 68.8 | 66.4 | 69 | 68.3 | 68.9 |
| Female | 32.6 | 32.4 | 30.9 | 31.2 | 33.6 | 31 | 31.7 | 31.1 |
| Patient's Census Region |  |  |  |  |  |  |  |  |
| Northeast | 28.7 | 21.8 | 25.4 | 28.7 | 24.5 | 18.8 | 25.5 | 34 |
| South | 44.4 | 43.3 | 31.7 | 28.8 | 46.9 | 43.6 | 33 | 29.3 |
| Midwest | 8.9 | 17.8 | 21 | 18.7 | 14.8 | 16.8 | 19.4 | 13.3 |
| West | 9.3 | 9.3 | 15.2 | 18.5 | 4.7 | 13.1 | 14.9 | 17.7 |
| Missing† | 8.9 | 7.8 | 6.6 | 5.4 | 9.1 | 7.7 | 7.2 | 5.7 |
| Area of Residence |  |  |  |  |  |  |  |  |
| Urban | 97.9 | 97.5 | 95.9 | 96 | 97.6 | 96.9 | 97.1 | 96.3 |
| Rural | 1 | 0.9 | 0.8 | 0.4 | 1.3 | 1.3 | 0.6 | 0.1 |
| Missing | 1.1 | 1.6 | 3.3 | 3.5 | 1.2 | 1.8 | 2.2 | 3.5 |
| Distance from patient to provider |  |  |  |  |  |  |  |  |
| <2 miles | 17.2 | 15.4 | 13.7 | 15.5 | 21.3 | 12.3 | 14.3 | 13.2 |
| 2-9 miles | 53.3 | 45.2 | 43.1 | 50.3 | 53.7 | 44.1 | 45.2 | 48.1 |
| 10-45 miles | 21.6 | 29.8 | 33.2 | 28 | 15.9 | 31.2 | 32.2 | 33.5 |
| >45 miles | 7.9 | 9.5 | 10 | 6.2 | 9 | 12.3 | 8.2 | 5.2 |
| Missing | 0.1 | 0 | 0 | 0 | 0.1 | 0 | 0 | 0 |
| Insurance Type or Primary Payor |  |  |  |  |  |  |  |  |
| Uninsured | 7.3 | 5.5 | 4.3 | 3.5 | 7.1 | 5.9 | 4.8 | 3.8 |
| Privately Insured | 22.3 | 27.7 | 30.8 | 34.2 | 20.8 | 25.8 | 30 | 35.6 |
| Medicaid | 30 | 22.3 | 16 | 9.8 | 31.3 | 21.1 | 18.1 | 12.1 |
| Medicare | 37.9 | 42.2 | 47 | 50.9 | 38.4 | 44.6 | 44.9 | 47.1 |
| Other Government | 0.8 | 1.1 | 1.1 | 0.7 | 0.9 | 1.3 | 1 | 0.6 |
| Missing | 1.6 | 1.1 | 0.9 | 0.9 | 1.5 | 1.2 | 1.3 | 0.8 |
| Cancer Care Facility Type |  |  |  |  |  |  |  |  |
| Community Cancer Program | 3.3 | 6.2 | 5.4 | 3.4 | 2.8 | 5.3 | 7.8 | 3.3 |
| Comprehensive Community Cancer Program | 21.5 | 26.4 | 33.9 | 34.8 | 21.7 | 31.2 | 28.7 | 32 |
| Academic/Research Program | 51.5 | 44.4 | 38.5 | 37.3 | 52.1 | 39.9 | 41.1 | 40.9 |
| Integrated Network Cancer Program | 14.9 | 15.3 | 15.6 | 19.2 | 14.4 | 15.9 | 15.3 | 18.1 |
| Missing† | 8.9 | 7.8 | 6.6 | 5.4 | 9.1 | 7.7 | 7.2 | 5.7 |

† Missing for those aged 40 years and below


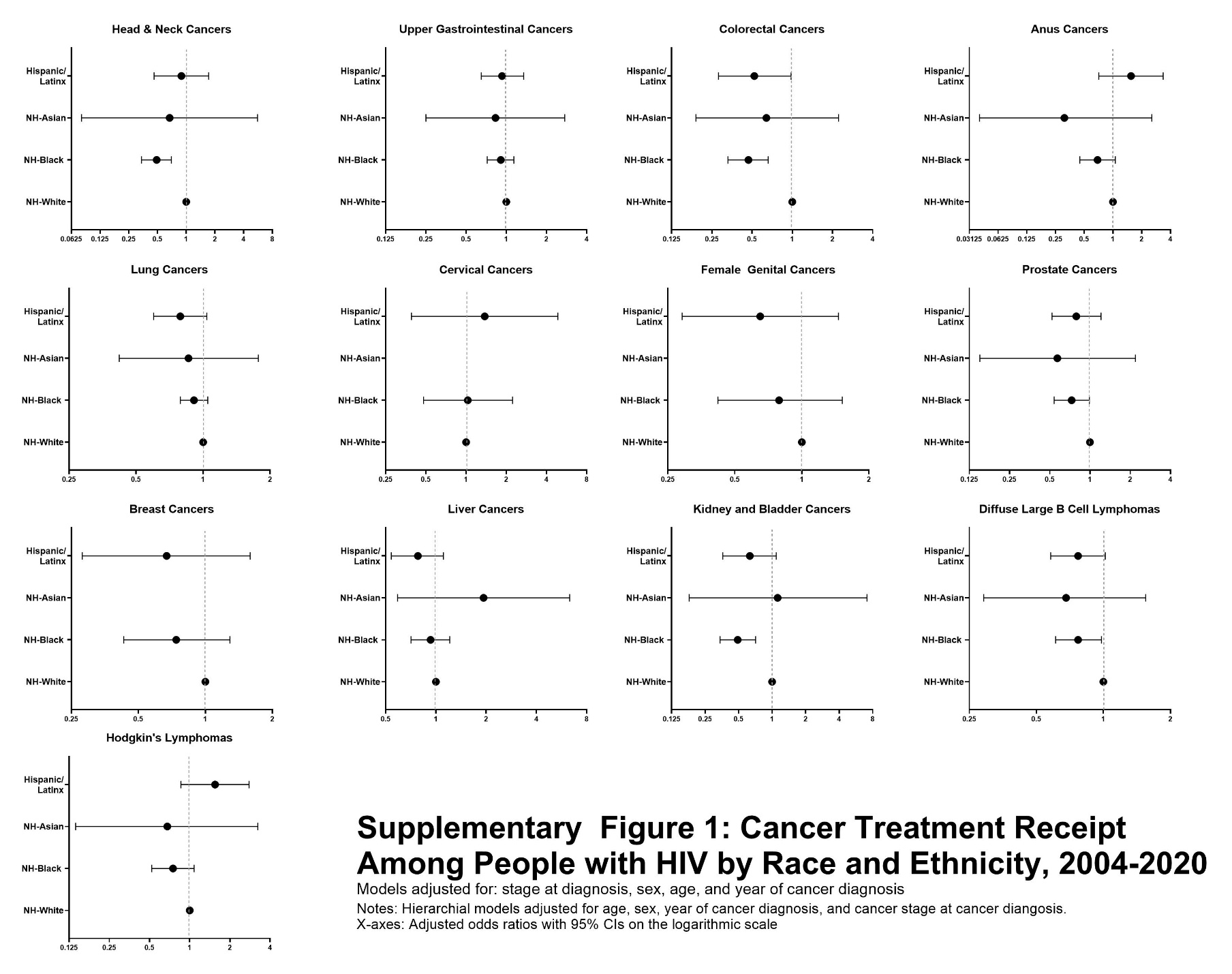

Supplement: Supplementary file 1 — Supplementary Material 1 [file 12885_2025_14272_MOESM1_ESM.docx]
